# The Crush: A Proto-Romantic Relationship Across the Life Course

**DOI:** 10.1093/geroni/igab046.1095

**Published:** 2021-12-17

**Authors:** Joseph Kotarba, Amanda Couve

**Affiliations:** 1 TxSt University, San Marcos, Texas, United States; 2 Encompass Health, San Antonio, Texas, United States

## Abstract

This presentation describes the “crush” experience as it occurs among older adults. A basic definition of a crush is a one-sided, proto-romantic relationship. The scholarly and commonsense understanding in American culture focuses on the crush as most commonly occurring during the developmental phases of adolescence and pre-adolescence. Symbolic interactionists view life course as a somewhat fluid process of adapting to changing situations in life. Experiences like the crush can potentially occur at almost any age at which romantic thoughts and feelings are possible. Our ethnographic research on older adults residing either in group facilities or in domiciliary locations indicates that crushes are fairly common. These crushes follow the same general narrative as crushes among younger people: a beginning, a middle and an end. There are two narrative styles among older adults: face-to-face and mediated. The crush in a group facility is encouraged by interaction during social hours, meals, entertainment, and religious/spiritual activities. Crushes are more observable among women who do not have to delve into their past for objects of their affection. Available paramours from the mass media include young celebrities such as Michael Buble and Josh Groban. These crushes differ from those among younger women in the denouement, to the degree affection generally fades away from memory rather than comes to a distinct end. Factors such as increased access to electronic media and music, and increased sociality in the community and in residential environments will create situations in which the security, excitement and rewards of a crush are plausible.

